# Retraction: Long Non-Coding RNA HOTAIR Regulates the Proliferation, Self-Renewal Capacity, Tumor Formation and Migration of the Cancer Stem-Like Cell (CSC) Subpopulation Enriched from Breast Cancer Cells

**DOI:** 10.1371/journal.pone.0300804

**Published:** 2024-03-26

**Authors:** The PLOS ONE Editors

Following the publication of this article [1], concerns were raised that images presented in Figs 2 and 4 appear similar to panels representing different experiments in articles [2–6] published by different author groups. Specifically:

○ Fig 2D LV-HOTAIR^KD^ in [1] and Fig 4C Transwell miR-99a of [2].
○ Fig 2D LV-scrambled in [1], Fig 3B H1299-miR-NC of [2], and Fig 6B 24h MG63-DDP/vector of [3].
○ Fig 2D LV-HOTAIR in [1] and Fig 4C Transwell miR-NC of [2].
○ Fig 2D LV-vector in [1] and Fig 6A 0h MG63/vector of [3].
○ Fig 4C LV-vector in [1] and Fig10A SK-HEP-1 shCCDC88A/VEGF of [4].
○ Fig 4C LV-HOTAIR in [1] and Fig 1A SNU-449 CSCs Mock of [5, retracted in 6].
○ Fig 4C LV-HOTAIR/miR-34a mimics in [1] and Fig 10A SK-HEP-1 shScrambled of [4].
○ Fig 4C LV-HOTAIR/scrambled mimics in [1] and Fig 10A SK-HEP-1 Mock of [4].

First author JD responded stating that they collaborated with authors of [2] and [3] at the Analysis Testing Center of Chengdu University of Traditional Chinese Medicine, and suggested that image errors may have occurred because research by these three groups was carried out around the same time using the same equipment and therefore images may have been misused. The Chengdu University of Traditional Chinese Medicine is neither listed among the affiliations of the authors of [1] nor mentioned in this article, and the explanation that the images may have been misused because different groups used the same equipment around the same time raises serious concerns about the overall data handling by the research groups. The underlying data provided by first author JD were insufficient to resolve the above concerns.

The above concerns call into question the reliability and provenance of the results. Therefore, the *PLOS ONE* Editors retract this article.

JD did not agree with the retraction. MY, RJ, NA, XW, and BL either did not respond directly or could not be reached.

Note: For panels in [1] that appear to overlap with the 2015 article [2], refer to the CC-BY 4.0 DEED license that applies to the original publication.
